# Genomic analysis of a *Raoultella ornithinolytica* strain causing prosthetic joint infection in an immunocompetent patient

**DOI:** 10.1038/s41598-018-27833-z

**Published:** 2018-06-21

**Authors:** Mamadou Beye, Issam Hasni, Piseth Seng, Caroline Michelle, Bernard La Scola, Didier Raoult, Pierre-Edouard Fournier

**Affiliations:** 1 Aix-Marseille Univ, IRD, AP-HM, SSA, VITROME, IHU Méditerranée-infection, Marseille, France; 2 Centre de Référence des Infections Ostéo-Articulaires (CRIOA) Sud-Méditerranée, Service des Maladies Infectieuses Chroniques, Pôle Maladies Infectieuses, Assistance Publique Hôpitaux de Marseille, Institut Hospitalo-Universitaire Méditerranée Infection, Marseille, France

## Abstract

We sequenced the genome of *Raoultella ornithinolytica* strain Marseille-P1025 that caused a rare case of prosthetic joint infection in a 67-year-old immunocompetent male. The 6.7-Mb genome exhibited a genomic island (RoGI) that was unique among *R. ornithinolytica* strains. RoGI was likely acquired by lateral gene transfer from a member of the *Pectobacterium* genus and coded for a type IVa secretion system found in other pathogenic bacteria and that may have conferred strain Marseille-P1025 an increased virulence. Strain Marseille-P1025 was also able to infect, multiply within, and kill *Acanthamoaeba castellanii* amoebae.

## Introduction

In 2001, the analysis of the 16S rRNA and *rpoB* gene sequences enabled reclassification of some *Klebsiella* species within the genus *Raoultella*^1^. Formerly known as *Klebsiella ornithinolytica*, *Raoultella ornithinolytica* is a Gram-negative, non-motile and encapsulated bacillus^1^ that inhabits aquatic environments and can also be found in hospital water circuits^2^. Reports of human *R. ornithinolytica* infections, initially rare, are increasing and mostly include biliary or urinary tract infections, and bacteremias^3–9^. Bone and joint infections caused by *R. ornithinolytica* are seldom reported^10^. We recently reported a case of chronic prosthetic joint infection caused by *R. ornithinolytica* in a 67-year-old immunocompetent male^11^. In this study, the causative strain, Marseille-P1025, was isolated from the peri-prosthetic pus^11^.

Herein, in order to determine whether this strain had specific virulence factors, we sequenced its genome and compared it to those of other *R. ornithinolytica* strains available in public databases.

## Results

### General genomic features

The draft genome sequence of *R. ornithinolytica* strain Marseille-P1025 consisted of 38 scaffolds after assembly and finishing. No putative plasmid sequence was detected. The chromosome size, G + C content, and CDS content were 5,644,584 bp, 55.6% and 5,260, respectively. A total of 86 RNA genes were identified, including one complete rRNA operon, a second 23S rRNA, eight other 5S rRNAs and 74 tRNAs. Of the 5,260 predicted CDSs, 4,391 genes were assigned a putative function (83.48%) and 869 (16.52%) were annotated as hypothetical proteins. A total of 4,438 (84.37%) genes were assigned a COG functional category.

### Genome comparison

**Figure 1:**
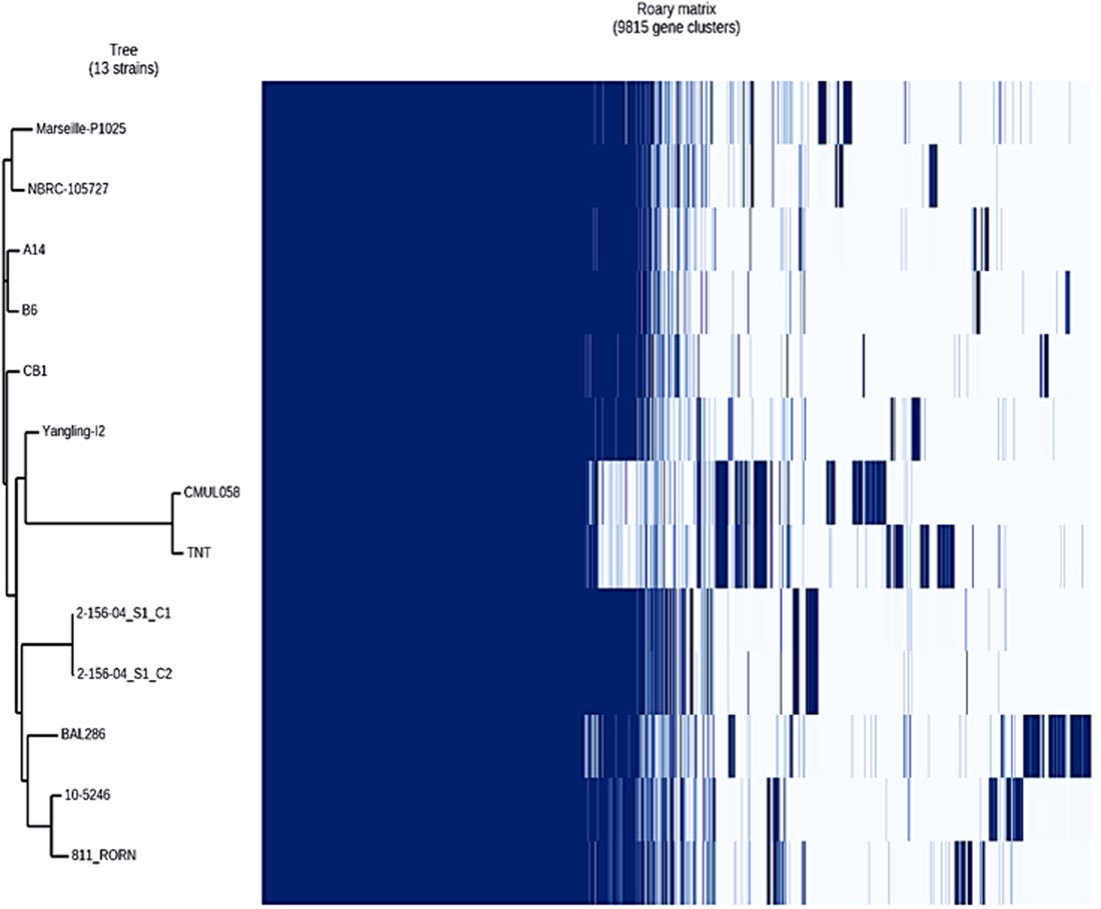
Pan-genome analysis of *R. ornithinolytica* whole-genome sequences. A maximum likelihood tree was constructed from the accessory genome elements (left). The presence (blue) and absence (white) of accessory genome elements is presented on the right.

The genomic comparison is summarized in Table 1. Strain Marseille-P1025, with 5,260 CDs, had a smaller genome than those of strains 10–5246, 2–156_04_S1_C1, 2-156-04_S1_C2, TNT, 811_RORN and BAL286 (5,288, 5,281, 5,284, 5,281, 5,314 and 5,646 CDs, respectively) but larger than those of strains NBRC 105727, B6, A14, CMUL058, CB1 and Yangling l2 (5,108, 4,907, 4,933, 5,202, 4,953 and 5,033 CDs, respectively) (Table 1). Strain Marseille-P1025 exhibited 95 specific genes (Fig. 1, Table 2) when compared to all other studied *R. ornithinolytica* strains. In contrast, 37 genes present in at least 7 strains were absent in strain Marseille-P1025 (Fig. 1, Table S1).

**Table 1:** Genomic comparison of *Raoultella ornithinolytica* strains.

Strains	GenBank accession numbers	Number of genes	Number of protein-coding genes	Number of RNAs	G + C content (%)
Strain Marseille-P1025	FTLF01000000	5,346	5,260	86	55.6
Strain 10–5246	AGDM00000000	5,353	5,288	65	55.5
Strain NBRC 105727	BCYR00000000	5,186	5,108	78	55.7
Strain B6	CP004142	5,018	4,937	81	55.9
Strain A14	CP008886	5,012	4,933	79	55.9
Strain CMUL058	CVRH00000000	5,288	5,202	86	55.7
Strain TNT	JHQH00000000	5,363	5,281	82	55.5
Strain 2-156- 04_S1_C1	JNPC00000000	5,380	5,281	99	55.6
Strain 2-156- 04_S1_C2	JNPD00000000	5,386	5,284	102	55.6
Strain 811_RORN	JURX00000000	5,332	5,314	18	55.6
Strain BAL286	JXXF00000000	5,706	5,646	60	55
Strain CB1	LFBW00000000	5,017	4,953	64	55.9
Strain Yangling I2	CP013338	5,125	5,033	92	55.7

**Table 2:** Functional annotation of the 95 specific genes of strain Marseille-P1025 among *R. ornithinolytica* strains.

Genes	Locus	Putative function (COGs category)
bicA_2	PROKKA_04516	sulfate permease (P)
can_2	PROKKA_04515	calcium ABC transporter ATPase (P)
cotSA	PROKKA_01597	Glycosyltransferase (M)
group_2403	PROKKA_02443	hypothetical protein (S)
group_5276	PROKKA_00062	hypothetical protein (S)
group_5277	PROKKA_00063	hypothetical protein (S)
group_5288	PROKKA_01595	Glycoside hydrolase (Not in Cogs)
mshA	PROKKA_01596	Glycosyltransferase (M)
group_5291	PROKKA_01598	hypothetical protein (S)
group_5295	PROKKA_01607	MATE efflux family protein, flippase (Not in Cogs)
group_5296	PROKKA_01608	Glycosyltransferase (G)
epsJ_2	PROKKA_01609	putative glycosyltransferase EpsJ (M)
group_5299	PROKKA_01612	pyruvyl transferase (M)
group_5302	PROKKA_01684	site-specific DNA-methyltransferase (L)
group_5305	PROKKA_01688	hypothetical protein (Not in Cogs)
group_5311	PROKKA_01727	competence CoiA-like family protein (R)
group_5312	PROKKA_01728	hypothetical protein (S)
group_5319	PROKKA_01736	molecular chaperone Tir (Not in Cogs)
group_5322	PROKKA_01739	hypothetical protein (Not in Cogs)
group_5332	PROKKA_02397	hypothetical protein (E)
group_5341	PROKKA_02408	integrating conjugative element protein (Not in Cogs)
group_5342	PROKKA_02409	carbamoyl transferase (Not in Cogs)
group_5343	PROKKA_02410	Glutamate synthase (Not in Cogs)
group_5344	PROKKA_02411	HNH endonuclease (Not in Cogs)
group_5345	PROKKA_02412	hypothetical protein (S)
group_5348	PROKKA_02415	Zinc-binding dehydrogenase. (Not in Cogs)
group_5349	PROKKA_02417	DNA adenine methylase (Not in Cogs)
intA_3	PROKKA_02432	Prophage CP4-57 integrase, Bacteriophage P4 integrase (L)
group_5360	PROKKA_02433	Prophage CP4-57 regulatory protein (AlpA) (K)
group_5361	PROKKA_02434	hypothetical protein (S)
group_5363	PROKKA_02436	integrase (Not in Cogs)
group_5365	PROKKA_02438	hypothetical protein (Not in Cogs)
group_5368	PROKKA_02441	DNA primase (R)
group_5369	PROKKA_02442	hypothetical protein (s).
group_5370	PROKKA_02564	hypothetical protein (S)
group_5371	PROKKA_02565	hypothetical protein (S)
group_5372	PROKKA_02566	hypothetical protein (S)
group_5382	PROKKA_02576	hypothetical protein (Not in Cogs)
group_5393	PROKKA_02589	Repressor (Not in Cogs)
group_5395	PROKKA_02591	hypothetical protein (Not in Cogs)
group_5396	PROKKA_02592	hypothetical protein (S)
ltrA	PROKKA_03114	Group II intron-encoded protein LtrA (X)
group_5405	PROKKA_03344	hypothetical protein (S)
group_5406	PROKKA_03345	hypothetical protein (S)
group_5407	PROKKA_03346	hypothetical protein (Not in Cogs)
group_5408	PROKKA_03347	Helix-turn-helix (E)
group_5409	PROKKA_03348	hypothetical protein (S)
group_5410	PROKKA_03349	tRNA_anti-like protein (Not in Cogs)
group_5412	PROKKA_04074	Reverse transcriptase (RNA-dependent DNA polymerase) (L)
group_5417	PROKKA_04503	hypothetical protein (Not in Cogs)
smc	PROKKA_04512	Chromosome partition protein Smc (R)
group_5422	PROKKA_04752	hypothetical protein (Not in Cogs)
group_5423	PROKKA_04753	hypothetical protein (Not in Cogs)
group_5424	PROKKA_04754	Integrase (L)
group_5426	PROKKA_05105	hypothetical protein (S)
group_5427	PROKKA_05106	hypothetical protein (Not in Cogs)
group_5429	PROKKA_05109	PemK-like protein (s)
group_5434	PROKKA_05117	DNA polymerase V subunit UmuC (Not in Cogs)
group_5440	PROKKA_05131	hypothetical protein (S)
**group_5441**	**PROKKA_05181**	**hypothetical protein (S)**
**group_5443**	**PROKKA_05184**	**hypothetical protein (S)**
**group_5444**	**PROKKA_05187**	**hypothetical protein (S)**
**group_5445**	**PROKKA_05188**	**hypothetical protein (S)**
**group_5446**	**PROKKA_05189**	**hypothetical protein (Not in Cogs)**
**group_5447**	**PROKKA_05190**	**hypothetical protein (Not in Cogs)**
**group_5448**	**PROKKA_05191**	**IS110 family transposase (X)**
**group_5450**	**PROKKA_05193**	**hypothetical protein (R)**
**traC_3**	**PROKKA_05194**	**DNA primase TraC (L)**
**group_5452**	**PROKKA_05195**	**cysteine desulfurase activator complex subunit SufD (Not in Cogs)**
**group_5453**	**PROKKA_05196**	**Type IV secretory system Conjugative DNA transfer (Not in Cogs)**
**group_5454**	**PROKKA_05197**	**conjugal transfer protein (Not in Cogs)**
**group_5455**	**PROKKA_05198**	**Type IV secretion system protein VirB11 (U)**
**group_5456**	**PROKKA_05199**	**Type IV secretion system protein virB10 (U)**
**virB9**	**PROKKA_05200**	**Type IV secretion system protein virB9 precursor (U)**
**ptlE**	**PROKKA_05201**	**Type IV secretion system protein PtlE (VirB8) (U)**
**group_5459**	**PROKKA_05202**	**Type IV secretion system proteins VirB7 (Not in Cogs)**
**group_5460**	**PROKKA_05203**	**TrbL/VirB6 plasmid conjugal transfer protein (U)**
**group_5461**	**PROKKA_05204**	**integrating conjugative element protein (Not in Cogs)**
**group_5462**	**PROKKA_05205**	**Type IV secretion system proteins (VirB5) (U)**
**virB4**	**PROKKA_05206**	**Type IV secretion system protein virB4(ATPase) (U)**
**group_5465**	**PROKKA_05209**	**hypothetical protein (Not in Cogs)**
**intA_5**	**POKKA_05210**	**Prophage CP4-57 integrase, Bacteriophage P4 integrase (L)**
group_5475	PROKKA_05235	hypothetical protein (Not in Cogs)
group_5483	PROKKA_05243	hypothetical protein (Not in Cogs)
group_5484	PROKKA_05244	hypothetical protein (Not in Cogs)
group_5485	PROKKA_05245	hypothetical protein (L)
group_5488	PROKKA_05248	hypothetical protein (S)
group_5493	PROKKA_05254	hypothetical protein (Not in Cogs)
group_5518	PROKKA_05291	hypothetical protein (S)
group_5536	PROKKA_05325	inner membrane protein (s)
group_5541	PROKKA_05330	hypothetical protein (Not in Cogs)
group_5542	PROKKA_05333	ATPase (D)
group_5543	PROKKA_05334	hypothetical protein (Not in Cogs)
group_5545	PROKKA_05338	hypothetical protein (Not in Cogs)
group_866	PROKKA_01605	hypothetical protein (Not in Cogs)

Genes from the RoGI genomic island are in bold.

The thirteen studied strains exhibited a pangenome and a core genome of 9,815 and 3,822 genes, respectively (Fig. 1). Figure 1 shows the dispersion of the pangenome of *R. ornithinolytica*. The phylogenetic analysis based on accessory genes clustered strains Marseille-P1025 and NBRC 105727.

### Functional annotation

**Figure 2:**
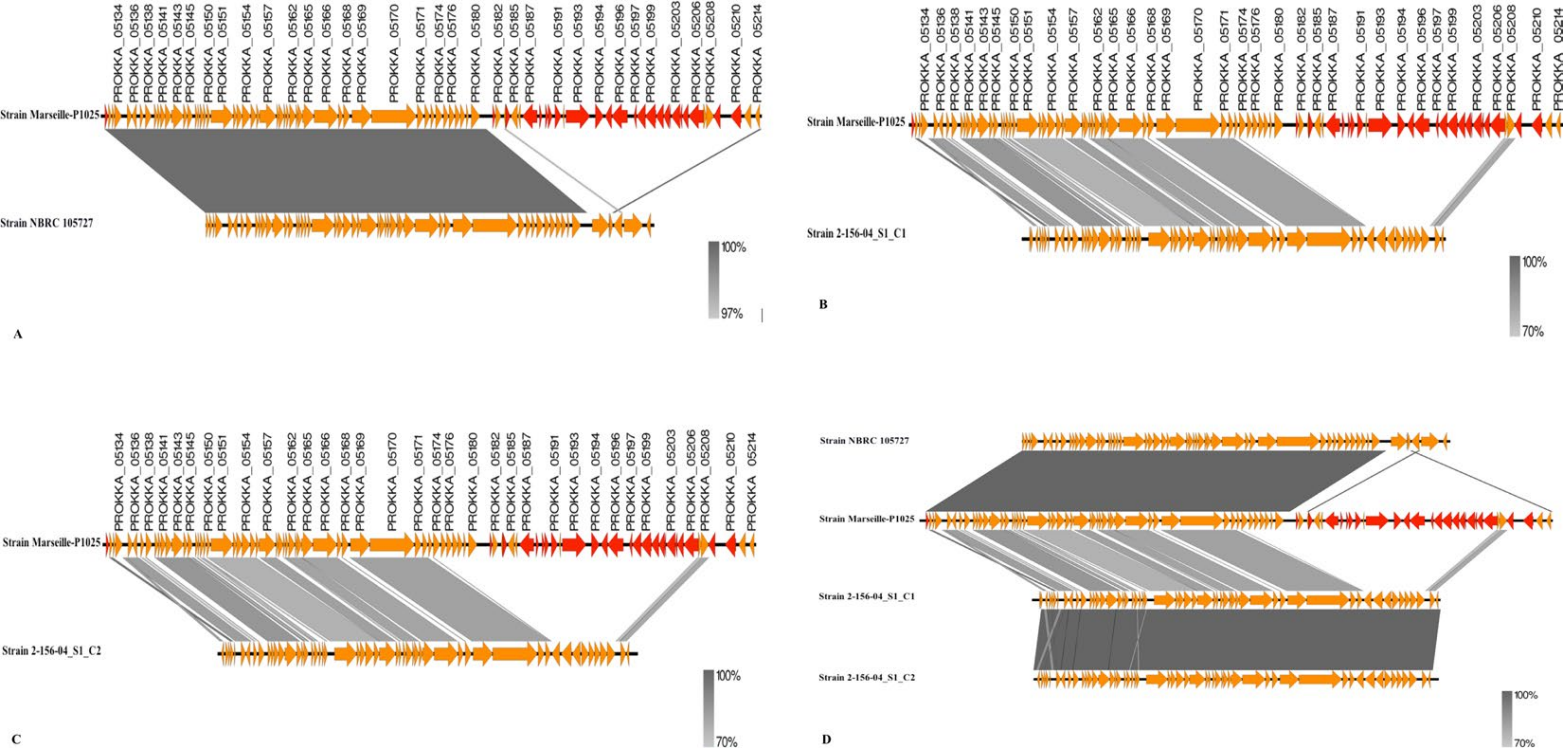
Comparison of sequences of the scaffold 21 from *R. ornithinolytica* strain Marseille-P1025 with those of *R. ornithinolytica* strains NBRC 105727 (**A**), 2-156-04_S1_C1 (**B**) and 2-156-04_S1_C2 (**C**). Figure 2D shows an alignment of all four compared genomes. Common and specific genes are displayed in orange and red, respectively.

The COG functional classification of the 95 genes specific of strain Marseille-P1025 demonstrated that 23 of the Marseille-P1025-specific genes were grouped in a 11,473-kb genomic island located in scaffold 21 (Fig. 2). This genomic island, which we named RoGI, exhibited a G + C content of 49.5% (*vs* 55 to 56% for the genomes of *R. ornithinolytica* strains, Table 1), and was absent from other *R. ornithinolytica* (Fig. 2). Of these 23 genes, nine coded type IVa secretion system proteins (Table 2), including seven VirB proteins (VirB 4 to 11, Table 2) and two proteins related to bacterial conjugation, including a type IVa secretion system conjugative DNA transfer protein and a conjugal transfer protein (Table 2). Moreover, the RoGI island contained a gene coding a second CP4-57 prophage integrase (intA) (Table 2). The genes coding the VirB1, VirB2, VirB3 and VirD4 proteins were identified at other locations of the genome from strain Marseille-P1025, thus supporting the assumption that this strain had a complete and putatively functional type IVa secretion system.

In addition, seven (7.4%) proteins were involved in intracellular trafficking and secretion, seven (7.4%) in replication and repair, four (4.2%) in cell wall/membrane/envelop biogenesis and four (4.2%) had a general functional prediction only (Table 2). Finally, three genes coded integrases including a CP4-57 prophage integrase (intA), two genes coded integrating conjugative element proteins, and two genes coded a CP4-57 prophage regulatory protein AlpA and a transposase, respectively (Table 2).

### ClonalframeML and Phylogenetic Analysis

**Figure 3:**
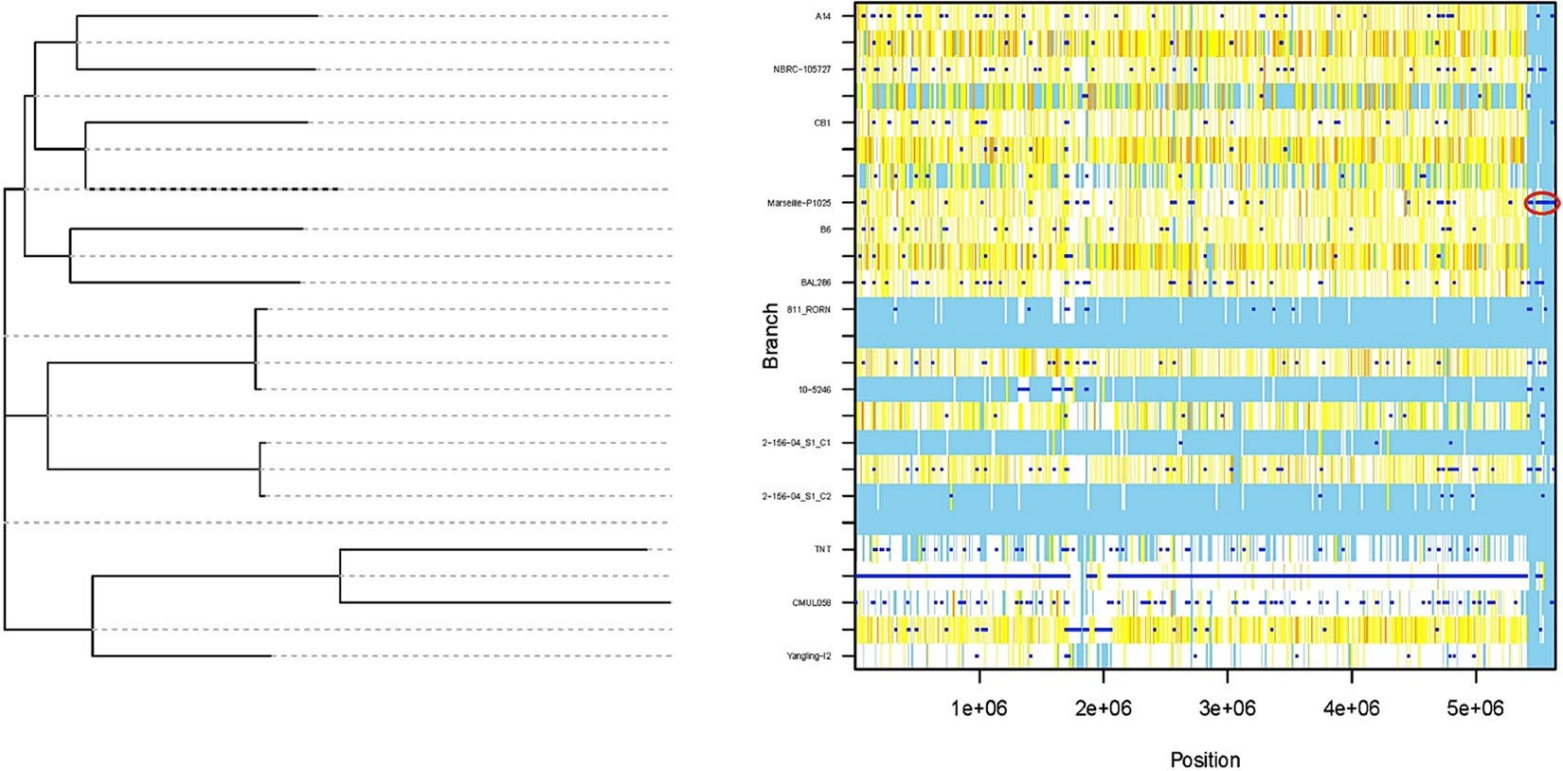
Analysis of genomic recombinations in the *R. ornithinolytica* species based on the alignment of 13 genomes including 12 genomes mapped against that of strain Marseille-P1025, using ClonalFrameML. Recombination events are shown by dark blue horizontal bars. For a given branch, light blue sites mean no substitution. Any other color from white to red indicates a substitution. White indicates non-homoplasic substitutions and the increasing level of redness indicates the increasing degree of homoplasy. The arrow shows recombination events in scaffold 21 where the RoGI genomic island is located.

To verify whether the RoGI island was acquired by lateral gene transfer, we used a recombination and phylogenetic analysis. Figure 3 shows the recombination events of external origin marked by a dark blue horizontal line. ClonalFrameML identified 170 recombination events on all branches of the clonal genealogy, including 23 recombination events in the genome of strain Marseille-P1025 (Fig. 3). These 23 regions appeared to be possible recombination hotspots (Fig. 3). Three of these recombination hotspots (red circle) were located in scaffold 21 of strain Marseille-P1025 (located from nucleotides 5,425,000 to 5,612,500) (Fig. 3), close to the RoGI island that coded the type IVa secretion system (located from nucleotides 5,504,317 to 5,515,790, Fig. 3).

The phylogenetic analysis of nucleotide sequences from the RoGI island supported the assumption that it was acquired by lateral gene transfer by identifying close phylogenetic neighbours in *Pectobacterium atrosepticum* strain JG10-08, *Pectobacterium sp*. strain SCC3193, two *Pectobacterium wasabiae* (strain CFBP-3304 and strain RNS08.42.1 A), *Cedecea neteri* strain ND14b and *Citrobacter amalonaticus* strain Y19 (Fig. S1).

### Conjugative pilus

**Figure 4:**
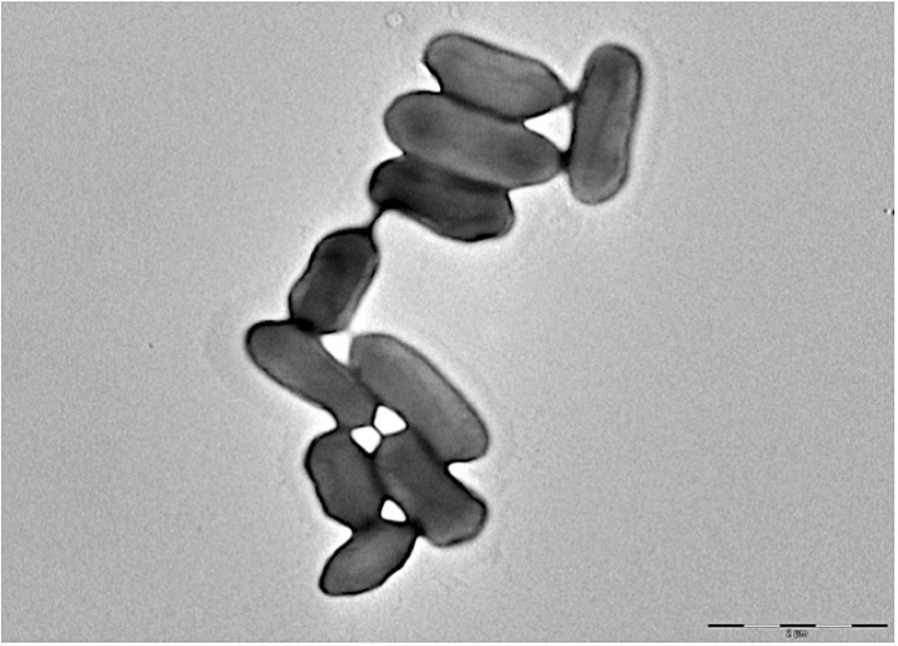
Transmission electron microscopy of *R. ornithinolytica* strain Marseille-P1025 using a Morgagni 268D transmission electron microscope (Philips) at an operating voltage of 60 kV. The scale bar represents 2 µm.

It is known that type IVa secretion systems elaborate pili to establish a host contact for substrate secretion or bacterial conjugation^12^. In order to confirm that strain Marseille-P1025 elaborates a conjugative pilus, electron microscopy was performed on cells after 24 h of incubation. Figure 4 shows that strain Marseille-P1025 possesses a conjugative pilus.

### Interaction of R. ornithinolytica with A. castellanii trophozoites

**Figure 5:**
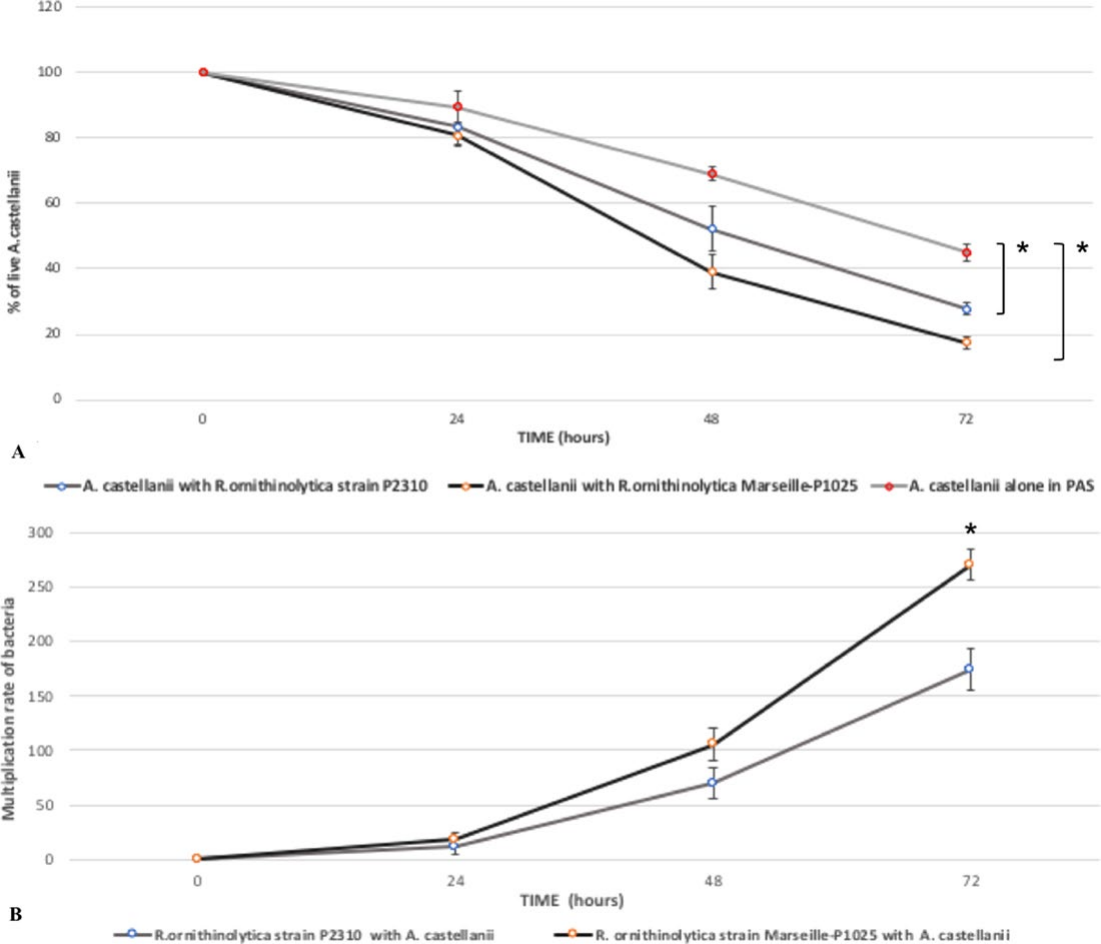
Co-culture of *R. ornithinolytica* and *A. castellanii* amoebae. (**A**) Rate multiplication of *Raoultella ornithinolytica* strains P2310 and Marseille-P1025 within *A*. *castellanii* in PAS at 32 °C. (**B**) Percentage of live *A*. *castellanii* infected with *R*. *ornithinolytica* strains P2310 and Marseille-P1025. Each bar represents the mean of triplicate wells, and the standard errors are represented by error bars. *P < 0.05.

**Figure 6:**
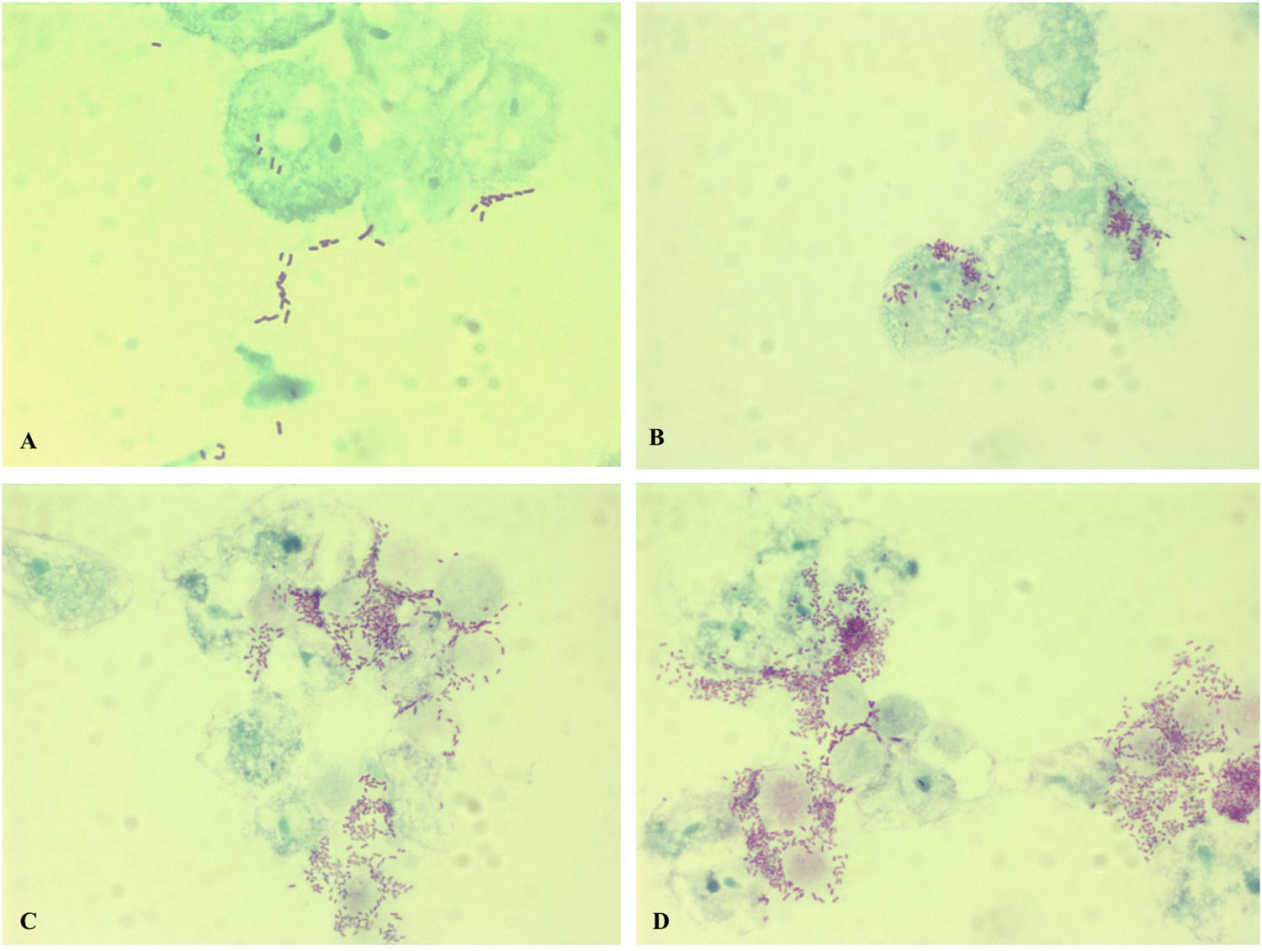
Optical microscopy observation of *A. castellanii* trophozoites infected with *R. ornithinolytica* strain Marseille-P1025 and stained with the Gimenez staining. The presence of *R. ornithinolytica* was monitored for 3 days: (**A**) day 0, co-culture after 5 hours of incubation; (**B**) day1, after 24 hours of incubation; (**C**) day 2, after 48 hours of incubation; (**D**) day 3, after 72 hours of incubation.

*Acanthamoeba castellanii* is a free-living amoeba that has previously been used as a eukaryote model to study the virulence of pathogenic microorganisms, including *Acinetobacter baumannii*, mycobacteria and streptococci^13–16^. To determine whether strain Marseille-P1025 can multiply in eukaryotic cells, triplicate co-culture assays were performed with *Acanthamoeba castellanii* amoebae. *Raoultella ornithinolytica* strain P2310, isolated from the feces of a healthy individual, was used as a control for this experiment (Figs 5 and 6). We observed that the numbers of both uninfected and infected *A. castellanii* trophozoites incubated into PAS at 32 °C decreased over time. However, the mean percentages of remaining live amoebae at day 3 were 45.0+/−1.33%, 19.69+/−1.44% and 27.83+/−4.82% for uninfected amoebae, amoebae infected with strain P2310 and amoebae infected with strain Marseille-P1025, respectively. Therefore, the number of infected amoebae decreased significantly more than those of uninfected amoebae (p < 0.05) in presence of both *R. ornithinolytica* strains (Figs 5A, S2), and strain Marseille-P1025 caused a higher amoebal mortality than strain P2310, although this difference was not statistically different (p = 0.17).

We also evaluated the numbers of CFUs obtained from intra-amoebal bacteria at H0 and H72 of co-culture (Figs 5B, S2). At H0 and H72, a mean 3.57 × 10^5^ CFUs/mL and 9.67 × 10^7^ CFUs/mL, respectively, were cultivated for strain Marseille-P1025 *versus* 5.73 × 10^5^ CFUs/mL and 9.33 × 10^7^ CFUs/mL, respectively, for strain P2310. The growth rate of both strains (270+/−97.5 and 174+/−29.3, respectively) was significantly higher for strain Marseille-P1025 (p < 0.05). Hence, these experiment demonstrated that strain Marseille-P1025 exhibited a higher pathogenicity for amoebae than the control strain (Fig. 5B). To confirm these observations, we examined bacteria within amoebae by Gimenez staining. Optical microscopy observations were consistent with the CFU evaluations. We observed that after 5 h of co-culture, most *A. castellanii* cells were infected by *R. ornithinolytica* strain Marseille-P1025 (Fig. 6A). Not only was *R. ornithinolytica* strain Marseille-P1025 able to survive within *A. castellanii*, but it began to multiply after 24 hours of co-culture (Fig. 6B). At day 2 of co-culture, strain Marseille-P1025 continued to multiply within amoebae. Furthermore, at day 3 of co-culture, infected amoebae started to lyse (Fig. 6D) whereas strain Marseille-P1025 kept multiplying. The lysis of *A. castellanii* amoebae was complete after 5 days of co-culture with bacteria (Fig. S3). We also observed that *R. ornithinolytica* survived in PAS medium without amoebae but did not multiply from day 0 to day 3.

## Discussion

Infections due to *R. ornithinolytica* are under-reported, possibly because this bacterium is difficult to identify using conventional phenotypic methods^17^. *Raoultella ornithinolytica* is currently regarded as an emerging hospital-acquired infection agent, particularly after invasive procedure^10^. Few pathogenic factors are recognized in *R. ornithinolytica* compared to other members of the family *Enterobacteriaceae*^10^. These include the ability to adhere to human tissues, to form biofilms in urinary catheters and to convert histidine to histamine in scombroid fishes, thus causing redness and flushing of the skin^10^.

By comparing the genome of strain Marseille-P1025 that had caused a chronic prosthetic joint infection in an immunocompetent patient, to those of other *R. ornithinolytica* strains, we identified a unique 11-kb genomic island (RoGI) among *R. ornithinolytica* strains. This genomic island coded nine proteins from the type IVa secretion system (SS) (Fig. 2, Table 2), four proteins related to bacterial conjugation and two integrases. In addition, the genome from strain Marseille-P1025 contained another four components of the type IVa SS, which suggests that this system was complete. The presence of a conjugative pili and the ability of strain Marseille-P1025 to invade, survive and multiply in an amoeba (*Acanthamoeba castellanii*) confirms the presence of type IVa secretion system.

The type IVa SS is involved in various mechanisms of bacterial pathogenesis such as the transfer of *Agrobacterium tumefaciens* oncogenic DNA into plants leading to tumours^18^. The Type IVa SS is also involved in conjugation and thus plays a crucial role in genomic plasticity, notably by enabling the transfer of plasmids carrying antibiotic resistance or virulence genes among pathogenic bacteria^19^. In addition, conjugation systems may also contribute significantly to the development of infections by promoting surface pili adhesion-mediated attachment, colonization and biofilm formation^20^. It is also reported that the type IVa secretion system, particularly the *virB* operon, is essential for survival and intracellular multiplication^21^. Siddiqui *et al*. have shown that pathogenic bacteria, but not those who are weakly or not pathogenic, can survive within *A. castellanii* cysts^16^. The ability of *R. ornithinolytica* to grow and cause cytopathic effect in *A. castellanii* seems to be correlated with its virulence^14,15^. We demonstrated that strain Marseille-P1025 was not only able to survive within amoebae but could also multiply more efficiently than the control strain and kill amoebae, supporting its virulent behavior.

We also demonstrated that the RoGI genomic island of strain Marseille-P1025 was closely related to sequences from the *Pectobacterium atrosepticum* strain JG10-08, *Pectobacterium* sp. strain SCC3193, two *Pectobacterium wasabiae* (strains CFBP-3304 and RNS08.42.1A), *Cedecea neteri* strain ND14b and *Citrobacter amalonaticus* strain Y19 (Fig. S1). As the genomes of *P. wasabiae* strains CFBP-3304 and RNS08.42.1 A, *P. atrosepticum* strain JG10-08 and *Pectobacterium sp*. strain SCC3193 exhibit genomic G + C contents of 50.6%, 50.4%, 51.1% and 50.4%, respectively, which is closer to that of the RoGI island (49.5%) than that of strain Marseille-P1025 (55.6%), we assume that it may have been transferred from a *Pectobacterium* or a phylogenetically-close species. *Pectobacterium* species (*P. wasabiae and P. atrosepticum*) are phytopathogens^22,23^ that belong to the *Enterobacteriaceae* family like *R. ornithinolytica*.

We also detected the presence of three specific genes carried by the CP4-57 prophage, including two integrases (*intA*) and *alpA*, a transcriptional regulator of *intA*^24^. The IntA integrase has been shown to intervene in biofilm formation. In *E. coli*, the deletion of the *intA* gene reduces early biofilm formation^24^ whereas the increased synthesis of IntA leads to excision of the CP4–57 prophage^24^ which, in turn, increases biofilm formation^24^. Therefore, IntA may play a role in biofilm formation in strain Marseille-P1025, thus facilitating its adhesion to foreign material such as the patient’s joint prosthesis.

In conclusion, *R. ornithinolytica* strain Marseille-P1025, that caused a rare case of chronic prosthetic joint infection in a 67-year-old immunocompetent male, exhibited a complete type IVa secretion system that was unique among *R. ornithinolytica* strains and was able to infect, multiply within, and kill amoebae. These properties may explain its particular virulence. In addition, this type IVa SS was mostly coded by a genomic island (RoGI) that was probably acquired by lateral gene transfer from *Pectobacterium* species.

## Material and Methods

### DNA extraction and Genome sequencing

Strain Marseille-P1025 was cultivated on Columbia agar (bioMérieux, Marcy-l’Etoile, France) at 37 °C in aerobic atmosphere for 24 hours. Then, after a pre-treatment with lysozyme at 37 °C for 2 hours, the DNA was extracted using an EZ1 biorobot and the EZ1 DNA tissue kit (Qiagen, hilden, Germany). The elution volume was 50 µL. Genomic DNA (gDNA) was quantified by a Qubit assay with the high sensitivity kit (Life technologies, Carlsbad, CA, USA) to 8 ng/µl, prior to being sequenced on a MiSeq sequencer (Illumina, San Diego CA, USA) with the Paired-End and barcode strategy in order to be mixed with 20 other projects constructed according to the Nextera XT library kit (Illumina).

One ng of gDNA was used as input and tagmented for the fragmentation step. Then, limited cycle PCR amplification completed the tag adapters and introduced dual-index barcodes. The libraries were then normalized on specific beads according to the Nextera XT protocol (Illumina), pooled into a single library and then loaded onto the reagent cartridge. Automated cluster generation and Paired-End sequencing with dual index reads was performed in a single 39-hour run in a 2 × 251-bp.

Total information of 9.8 Gb was obtained from a 1,165 K/mm 2 cluster density with 88% (18,993,000 clusters) of the clusters passing quality control filters. Within this pooled run, the index representation of *R. ornithinolytica* strain Marseille-P1025 was determined to be 5.51%. The 1,046,713 Paired-End reads were filtered according to the read qualities.

### Genome annotation and comparison

The sequencing reads were assembled using the A5 assembler^25^. Then, a step of finishing was done using the Mauve software^26^ and CLC bioserver. After assembly and finishing, the genome size was 5.6 Mb. Open reading frames (ORFs) were predicted using the Prodigal tool (http://prodigal.ornl.gov) with defaults parameters. The prediction of protein function was performed by searching against the GenBank database using BLASTP algorithm^27^. Functional classification of gene families (COG ID and Letters) was obtained using COGnitor against the COG database^28^. tRNAs and rRNAs were detected using tRNAscan-SE v.1.21^29^ and RNAmmer v.1.2^30^, respectively. The presence or absence of plasmids was verified both by searching the gene annotation for any plasmid-related gene and by mapping all contigs against previously published *Raoultella* sp. plasmid sequences.

We compared the genome sequence of *R. ornithinolytica* strain Marseille-P1025 to those of other strains of this species found in public databases. As of August 30^th^, 2016 13 *R. ornithinolytica* genomes were available in public databases. Of these, we used 12 genomes for comparative analysis and excluded that of strain S12 due to its insufficient quality. The twelve comparator genomes were those from strains 10-5246 (AGDM00000000), NBRC 105727 (BCYR00000000), B6 (CP004142), A14 (CP008886), CMUL058 (CVRH00000000), TNT (JHQH00000000), 2-156-04_S1_C1 (JNPC00000000), 2-156-04_S1_C2 (JNPD00000000), 811_RORN (JURX00000000), BAL286 (JXXF00000000), CB1 (LFBW00000000) and Yangling I2 (CP013338). All genomes were re-annotated using the Prokka software, version 1.11^31^. Comparisons between all selected genomes were done using Roary, a tool that rapidly builds large-scale pangenomes^32^, with a blast identity cut-off of 97% for the comparison between *R. ornithinolytica* species. In addition, Roary identified the specific and missing genes from strain Marseille-P1025. Specific genes were checked by BLASTP and TBLASTN against the other studied genomes. Missing genes were checked by TBLASTN against the genome of strain Marseille-P1025, using a coverage and identity of 60% and 80% as thresholds, respectively, as described by Kuenne *et al*.^33,34^. Easyfig.^35^ was used to visualise the coding regions and colour the specific genes of strain Marseille-P1025.

### Recombination and Phylogenetic analysis

The genome of strain Marseille-P1025 was used as a reference for whole-genome alignment^36^ using Mugsy^37^. Then, a phylogenetic tree based on whole genome sequence alignment was done using the FastTree software^38^ and the maximum likelihood method (Fig. 1). ClonalFrameML was used to search recombination hotspots in *R. ornithinolytica* genomes by analyzing both the whole genome alignment and the phylogenetic tree^39^.

Unique sequences were detected by a BLASTN search for homologous sequences and multiple sequence alignment using the Mafft software algorithm^40^. Phylogenetic analysis of these unique sequences was performed using MEGA version 7^41^ and the maximum likelihood (ML) algorithm, with 1,000 bootstrap replicates.

### Electron microscopy

Electron microscopy was performed with detection Formvar coated grids. Forty 40 μL of bacterial suspension were deposited on a grid and incubated at 37 °C for 30 min, followed by a 10 sec incubation on ammonium molybdate 1%. Grids were then observed using a Morgagni 268D transmission electron microscope (Philips) at an operating voltage of 60 kV.

### Culture of R. ornithinolytica and A. castellanii

*Raoultella ornithinolytica* strain P2310, isolated from the feces of a healthy individual, was used as a control in co-culture experiments. *Raoutella ornithinolytica* strains Marseille-P1025 and P2310 were grown on 5% sheep blood-enriched Columbia agar (BioMérieux) at 35 °C for 24 hours in anaerobic atmosphere. Bacteria were then harvested, centrifuged at 4,000 × g during 5 minutes, washed twice and suspended in Page’s modified Neff’s amoeba saline (PAS). The PAS medium was prepared as follows: solution A (for 100 mL of sterile distilled water), 1.2 g NaCl + 0.04 g MgSO_4_.7H_2_O + 1.42 g Na_2_HPO_4_ + 1.36 g KH_2_PO_4_; solution B (for 100 mL of sterile distilled water), 0.04 g CaCl_2_.2H_2_O; PAS solution −10 mL of solution A + 10 mL of solution B + 980 mL of sterile distilled water). The inoculum density was determined by the McFarland method.

*Acanthamoeba castellanii* strain Neff (ATCC 30010) was grown in 175 cm² culture flasks containing 30 mL peptone-yeast extract-glucose (PYG) at 28 °C. When a monolayer was formed, *A. castellanii* trophozoites were harvested by shaking the flasks and centrifuged at 500 × g for 10 min. The pellet was suspended in 30 mL PAS medium. The quantification of the *A. castellanii* population was performed using a KOVA® slide cell counting chamber.

### Co-culture experiments

The amoebal trophozoite suspension (5 × 10^5^ amoeba/mL) was inoculated in 24-well plates and allowed to adhere for 30 minutes at 32 °C. Then, *R. ornithinolytica* suspensions were inoculated on amoebae to achieve ratios of infection of 10 *R. ornithinolytica* cells/amoeba. As controls, *A. castellanii* and *R. ornithinolytica* strains were cultivated separately in PAS. After incubation for 2.5 h at 32 °C under a 5% CO_2_ atmosphere, the co-culture wells were washed three times with PAS to remove any remaining extracellular or adherent bacteria. Incubation at 32 °C under 5% CO_2_ was then performed for 3 days. The presence of viable *Raoultella* inside amoebal trophozoites was documented by sub-culturing at 0, 24, 48 and 72 h of incubation. For each time point, the *A. castellanii* monolayer from a well was lysed by three passages through a 25-gauge needle. Serial dilutions of the lysate were carried out, plated onto COS medium and incubated for 2 days at 32 °C under anaerobic atmosphere to determine the numbers of intracellular *R. ornithinolytica* colony forming units (CFU). Multiplication rate of the bacterial invasion was calculated as follows: recovered *R. ornithinolytica* (CFU)/*R. ornithinolytica* (CFU) at time 0. The *A. castellanii* population was also monitored during the 3-day experiment: counting and viability check of amoebae, cultivated alone and in co-culture, was performed using KOVA® slides after Trypan Blue 0.4% coloration (Sigma-Aldrich, Taufkirchen, Germany). All experiments were reproduced three times, each time in duplicate. The standard error of the mean (SEM) was used to evaluate the experiment value distribution. To compare the intra-amoebal growth of the two tested bacterial strains, we also estimated the dayly multiplication rate of bacteria.

The presence of *R. ornithinolytica* within amoebae was also monitored for 5 days by Gimenez staining^42^. The observation was performed with a LEICA DM 2500 LED microscope.

### Statistical analyses

Statistical analyses mentioned in this study were performed using the Student’s t-test and Chi-square test, with a significance level of *P* inferior or equal to 0.05.

### Nucleotide sequence accession numbers

The genome sequence from *R. ornithinolytica* strain Marseille-P1025 was deposited in GenBank under accession number FTLF01000000.

## Electronic supplementary material

Supplementary data

## References

[1] Drancourt M, Bollet C, Carta A, Rousselier P (2001). Phylogenetic analyses of *Klebsiella* species delineate *Klebsiella* and *Raoultella* gen. nov., with description of *Raoultella ornithinolytica* comb. nov., *Raoultella terrigena* comb. nov. and *Raoultella planticola* comb. nov. Int. J. Syst. Evol. Microbiol..

[2] Kanki M, Yoda T, Tsukamoto T, Shibata T (2002). *Klebsiella pneumoniae* produces no histamine: *Raoultella planticola* and *Raoultella ornithinolytica* strains are histamine producers. Appl. Environ. Microbiol..

[3] Hadano Y (2012). *Raoultella ornithinolytica* bacteremia in cancer patients: report of three cases. Intern. Med..

[4] Haruki Y (2014). Clinical characteristics of *Raoultella ornithinolytica* bacteremia: A case series and literature review. J. Infect. Chemother..

[5] Mau N, Ross LA (2010). *Raoultella ornithinolytica* bacteremia in an infant with visceral heterotaxy. Pediatr. Infect. Dis. J..

[6] Chun S, Yun JW, Huh HJ, Lee NY (2014). Clinical characteristics of *Raoultella ornithinolytica* bacteremia. Infection.

[7] Sibanda, M. Primary peritonitis caused by *Raoultella ornithinolytica* in a 53‐year‐old man. *JMM Case Rep*. **1**, (2014).

[8] Cleveland KO, Mazumder SA, Gelfand MS (2014). Association of *Raoultella* bacteremia with diseases of the biliary tract. Scand. J. Infect. Dis..

[9] Jong Ede (2014). Predominant association of *Raoultella* bacteremia with diseases of the biliary tract. Scand. J. Infect. Dis..

[10] Seng P (2016). Emerging role of *Raoultella ornithinolytica* in human infections: a series of cases and review of the literature. Int. J. Infect. Dis. IJID Off. Publ. Int. Soc. Infect. Dis..

[11] Seng P (2016). *Raoultella ornithinolytica*: An unusual pathogen for prosthetic joint infection. IDCases.

[12] Darbari VC, Waksman G (2015). Structural biology of bacterial type IV secretion systems. Annu. Rev. Biochem..

[13] Da Silva JL, Nguyen J, Fennelly KP, Zelazny AM, Olivier KN (2018). Survival of pathogenic *Mycobacterium abscessu*s subsp. massiliensein Acanthamoeba castellanii. Res. Microbiol..

[14] Tamang MD, Kim S, Kim S-M, Kong H-H, Kim J (2011). Interaction of *Acinetobacter baumannii* 19606 and 1656-2 with *Acanthamoeba castellanii*. J. Microbiol..

[15] Goy G (2007). The Neff strain of *Acanthamoeba castellanii*, a tool for testing the virulence of *Mycobacterium kansasii*. Res. Microbiol..

[16] Siddiqui R, Lakhundi S, Khan NA (2015). Interactions of *Pseudomonas aeruginosa* and *Corynebacterium* spp. with non-phagocytic brain microvascular endothelial cells and phagocytic *Acanthamoeba castellanii*. Parasitol. Res..

[17] Park JS (2011). Evaluation of three phenotypic identification systems for clinical isolates of *Raoultella ornithinolytica*. J. Med. Microbiol..

[18] McCullen CA, Binns AN (2006). *Agrobacterium tumefaciens* and plant cell interactions and activities required for interkingdom macromolecular transfer. Annu. Rev. Cell Dev. Biol..

[19] Juhas M (2007). Novel type IV secretion system involved in propagation of genomic islands. J. Bacteriol..

[20] Gonzalez-Rivera, C., Bhatty, M. & Christie, P. J. Mechanism and function of type IV secretion during infection of the human host. *Microbiol. Spectr*. **4**, (2016). 10.1128/microbiolspec.VMBF-0024-2015 PMC4920089 27337453

[21] Sieira R, Comerci DJ, Sánchez DO, Ugalde RA (2000). A homologue of an operon required for DNA transfer in *Agrobacterium* is required in *Brucella abortus* for virulence and intracellular multiplication. J. Bacteriol..

[22] Nykyri, J. *et al*. Revised phylogeny and novel horizontally acquired virulence determinants of the model soft rot phytopathogen *Pectobacterium wasabiae* SCC3193. *PLoS Pathog*. **8**, (2012). 10.1371/journal.ppat.1003013 PMC3486870 23133391

[23] De Boer SH, Li X, Ward LJ (2012). *Pectobacterium* spp. associated with bacterial stem rot syndrome of potato in Canada. Phytopathology.

[24] Wang X, Kim Y, Wood TK (2009). Control and benefits of CP4-57 prophage excision in *Escherichia coli* biofilms. ISME J..

[25] Tritt A, Eisen JA, Facciotti MT, Darling AE (2012). An Integrated pipeline for de novo assembly of microbial genomes. PLoS ONE.

[26] Darling ACE, Mau B, Blattner FR, Perna NT (2004). Mauve: Multiple alignment of conserved genomic sequence with rearrangements. Genome Res..

[27] Altschul, S. F. BLAST Algorithm. in *eL*S(ed. John Wiley & Sons Ltd). 10.1002/9780470015902.a0005253.pub2 (John Wiley & Sons, Ltd, 2014).

[28] Tatusov RL, Galperin MY, Natale DA, Koonin EV (2000). The COG database: a tool for genome-scale analysis of protein functions and evolution. Nucleic Acids Res..

[29] Lowe TM, Eddy SR (1997). tRNAscan-SE: A program for improved detection of transfer RNA genes in genomic sequence. Nucleic Acids Res..

[30] Lagesen K (2007). RNAmmer: consistent and rapid annotation of ribosomal RNA genes. Nucleic Acids Res..

[31] Seemann T (2014). Prokka: rapid prokaryotic genome annotation. Bioinformatics.

[32] Page, A. J. *et al*. Roary: rapid large-scale prokaryote pan genome analysis. *Bioinforma. Oxf. Engl*. 10.1093/bioinformatics/btv421 (2015). 10.1093/bioinformatics/btv421 PMC4817141 26198102

[33] Rychli, K. *et al*. Genome sequencing of *Listeria monocytogenes* ‘Quargel’ listeriosis outbreak strains reveals two different strains with distinct *in vitro* virulence potential. *PLoS ONE* **9**, (2014). 10.1371/journal.pone.0089964 PMC3935953 24587155

[34] Kuenne C (2013). Reassessment of the *Listeria monocytogenes* pan-genome reveals dynamic integration hotspots and mobile genetic elements as major components of the accessory genome. BMC Genomics.

[35] Sullivan MJ, Petty NK, Beatson SA (2011). Easyfig: a genome comparison visualizer. Bioinformatics.

[36] Maiden MCJ (2013). MLST revisited: the gene-by-gene approach to bacterial genomics. Nat. Rev. Microbiol..

[37] Angiuoli SV, Salzberg SL (2011). Mugsy: fast multiple alignment of closely related whole genomes. Bioinformatics.

[38] Price MN, Dehal PS, Arkin AP (2009). FastTree: computing large minimum evolution trees with profiles instead of a distance matrix. Mol. Biol. Evol..

[39] Didelot, X. & Wilson, D. J. ClonalFrameML: efficient inference of recombination in whole bacterial genomes. *PLoS Comput. Biol*. **11**, (2015). 10.1371/journal.pcbi.1004041 PMC4326465 25675341

[40] Katoh K, Standley DM (2013). MAFFT multiple sequence alignment software version 7: improvements in performance and usability. Mol. Biol. Evol..

[41] Tamura K, Stecher G, Peterson D, Filipski A, Kumar S (2013). MEGA6: Molecular Evolutionary Genetics Analysis version 6.0. Mol. Biol. Evol..

[42] Giménez DF (1964). Staining rickettsiae in yolk-sac cultures. Stain Technol..

