# Supplementary material for: Genomic analysis of a Raoultella ornithinolytica strain causing prosthetic joint infection in an immunocompetent patient
Source: Sci Rep. 2018 Jun 21;8:9462. doi: 10.1038/s41598-018-27833-z (PMC6013458; doi:10.1038/s41598-018-27833-z)
Supplement: Supplementary file 1 — Supplementary data [file 41598_2018_27833_MOESM1_ESM.docx]

**Genomic analysis of a *Raoultella ornithinolytica* strain causing prosthetic joint infection in an immunocompetent patient**

Mamadou Beye^1^, Issam Hasni^1^, Piseth Seng^1, 2^, Caroline Michelle^1^, Bernard La Scola^1^, Didier Raoult^1^ and Pierre-Edouard Fournier^1, 2^

^1^ Aix-Marseille Université, URMITE, UM63, CNRS7278, IRD198, Inserm1095, Assistance Publique-Hôpitaux de Marseille, Institut Hospitalo-Universitaire Méditerranée-infection, Marseille, France.

^2^ Centre de Référence des Infections Ostéo-Articulaires (CRIOA) Sud-Méditerranée, Service des Maladies Infectieuses Chroniques, Pôle Maladies Infectieuses, Assistance Publique Hôpitaux de Marseille, Institut Hospitalo-Universitaire Méditerranée Infection, Marseille, France

* Corresponding author:

Pierre-Edouard Fournier

URMITE, IHU Méditerranée-Infection

19-21 Bd Jean Moulin

13005 France

Tel: + 33 413 732 401; fax: +33 413 732 402

pierre-edouard.fournier@univ-amu.fr

Keywords: *Raoultella ornithinolytica*; prosthetic joint infection; bone and joint infection; genome analysis; type IVa secretion system; bacteria; human.

**Supplementary Files:**

**Figure S1:**

Phylogenetic tree showing the position of *Raoultella ornithinolytica* strain Marseille-P1025 among other bacteria using sequences of the RoGI genomic island and the maximum likelihood method. Percentages at the nodes correspond to bootstrap values obtained from 1,000 replicates. Only values greater than 70% are displayed.

**Figure S2:**

Optical microscopy observation of lysed *A. castellanii* trophozoites infected with *R. ornithinolytica* strain Marseille-P1025 and stained by the Gimenez method after 5 days of co-culture.

**Table S1**: Functional annotation of genes absent from strain Marseille-P1025 but present in other *R*. *ornithinolytica* genomes. Present genes are marked by black cells whereas white cells indicate genes that are absent.


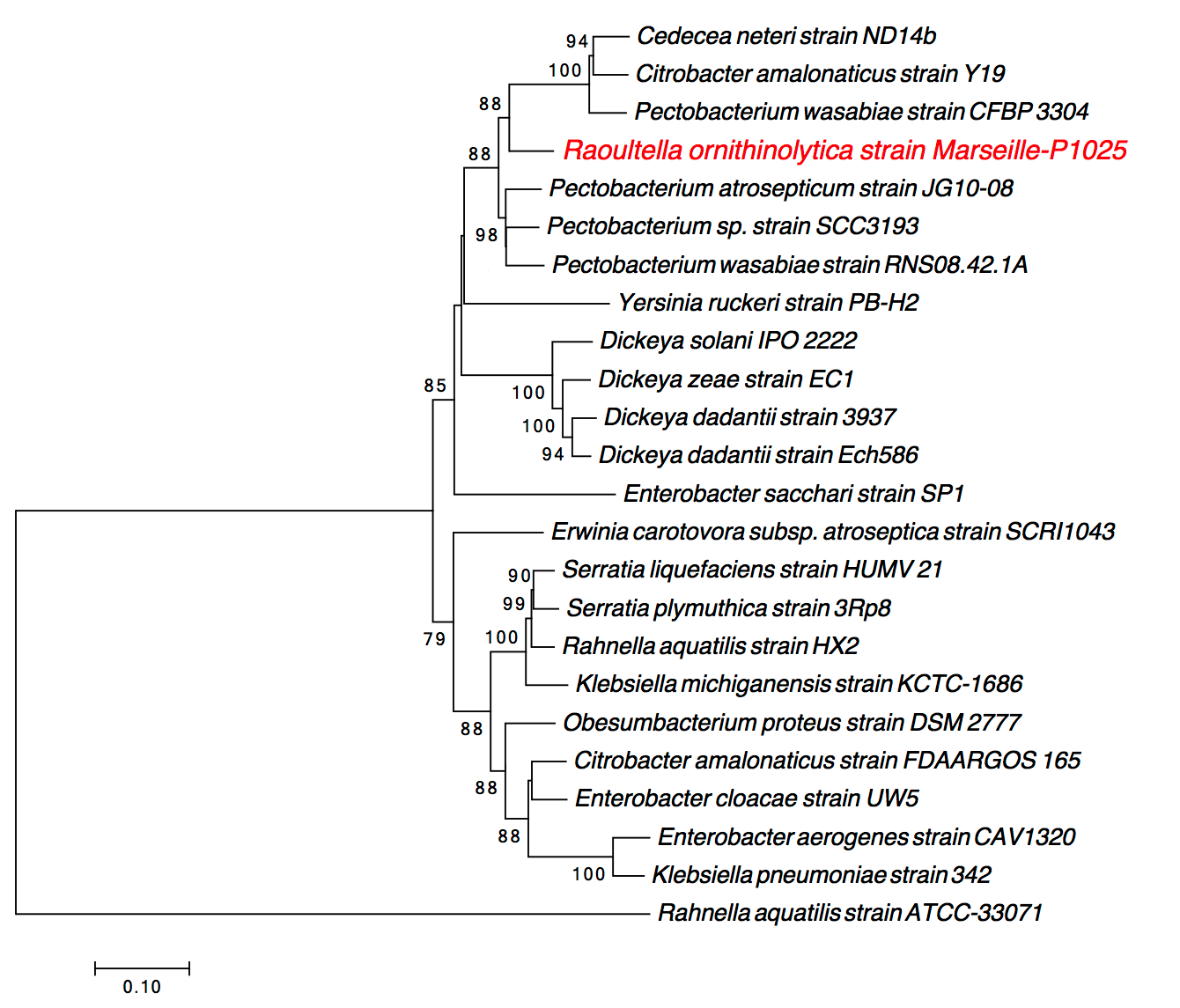


**Figure S1**: Phylogenetic tree showing the position of *Raoultella ornithinolytica* strain Marseille-P1025 among other bacteria using sequences of the RoGI genomic island and the maximum likelihood method. Percentages at the nodes correspond to bootstrap values obtained from 1,000 replicates. Only values greater than 70% are displayed.


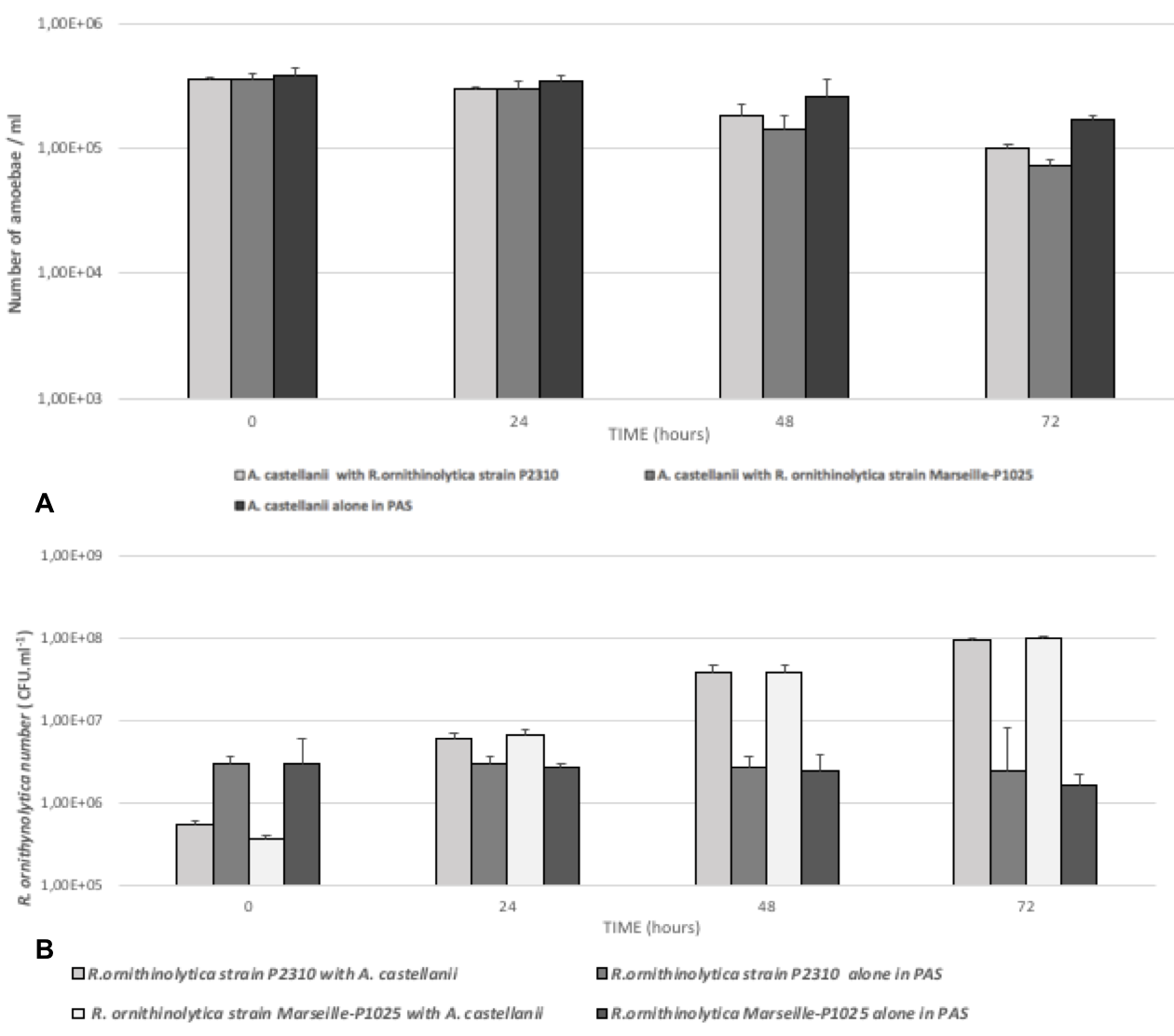


Figure S2: **Co-culture of *R. ornithinolytica* and *A. castellanii* amoebae***.* **A**: Amoebal viability in PAS at 32°C, in presence (dark gray bar and light gray bar) or absence (black bar) of *R.ornithinolytica*. Each bar represents the mean of triplicate wells, and the standard errors are represented by error bars. **B:** Growth of *R. ornithinolytica* in PAS medium at 32°C without (dark gray bar and black bar) or within *A. castellanii* trophozoites (light gray bar and white bar). Each bar represents the mean of triplicate wells, and the standard errors are represented by error bars.


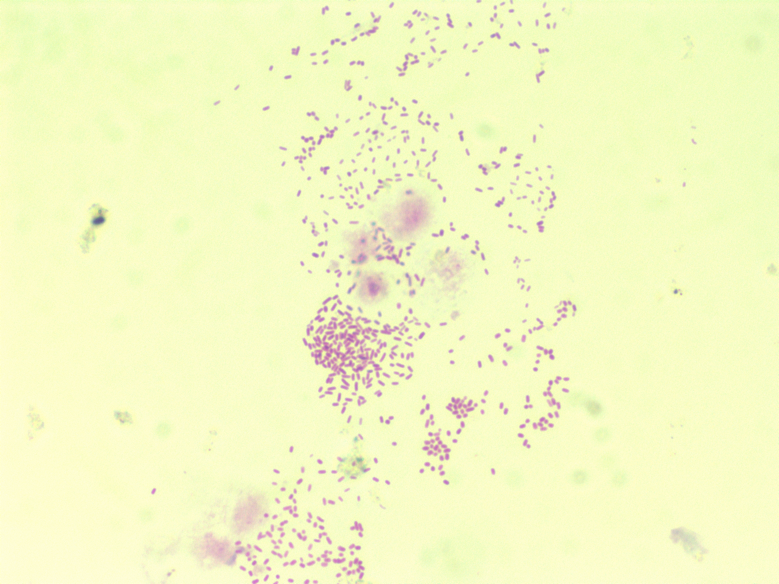


**Figure S3**: Optical microscopy observation of lysed *A. castellanii* trophozoites infected with *R. ornithinolytica* strain Marseille-P1025 and stained by the Gimenez method after 5 days of co-culture.

**Table S1**: Functional annotation of genes absent from strain Marseille-P1025 but present in other *R*. *ornithinolytica* genomes. Present genes are marked by black cells whereas white cells indicate genes that are absent.

| **Gene** | **Putative function (COGs category)** | **Number of isolates** | **10-5246** | **NBRC 105727** | **B6** | **A14** | **Yangling I2** | **CMUL058** | **TNT** | **2-156- 04_S1_C1** | **2-156- 04_S1_C2** | **811_RORN** | **BAL286** | **CB1** |
| --- | --- | --- | --- | --- | --- | --- | --- | --- | --- | --- | --- | --- | --- | --- |
| pcaK_2 | MFS transporter [G] | 12 |  |  |  |  |  |  |  |  |  |  |  |  |
| araE_1 | Arabinose-proton symporter [G] | 11 |  |  |  |  |  |  |  |  |  |  |  |  |
| fucK_1 | carbohydrate kinase [G] | 11 |  |  |  |  |  |  |  |  |  |  |  |  |
| group_1453 | putative FAD-linked oxidoreductase [C] | 11 |  |  |  |  |  |  |  |  |  |  |  |  |
| group_1454 | hypothetical protein (Not in CoGs) | 11 |  |  |  |  |  |  |  |  |  |  |  |  |
| group_2109 | 6-N-hydroxylaminopurine resistance protein (s) | 11 |  |  |  |  |  |  |  |  |  |  |  |  |
| lacC_2 | Tagatose-6-phosphate kinase [G] | 11 |  |  |  |  |  |  |  |  |  |  |  |  |
| lsrF_1 | putative aldolase Ls [G] | 11 |  |  |  |  |  |  |  |  |  |  |  |  |
| rbsD_1 | carbohydrate transporter [G] | 11 |  |  |  |  |  |  |  |  |  |  |  |  |
| xylE | MFS sugar transporter or D-xylose-proton symporter [G] | 11 |  |  |  |  |  |  |  |  |  |  |  |  |
| dmlR_27 | HTH-type transcriptional regulator DmlR [K] | 10 |  |  |  |  |  |  |  |  |  |  |  |  |
| dmpI | 2-hydroxymuconate tautomerase [R] | 10 |  |  |  |  |  |  |  |  |  |  |  |  |
| uxuA_2 | Mannonate dehydratase [G] | 10 |  |  |  |  |  |  |  |  |  |  |  |  |
| asr | Acid shock protein [M] | 9 |  |  |  |  |  |  |  |  |  |  |  |  |
| group_1788 | hypothetical protein [S] | 8 |  |  |  |  |  |  |  |  |  |  |  |  |
| group_2191 | hypothetical protein (Not in CoGs) | 8 |  |  |  |  |  |  |  |  |  |  |  |  |
| group_3642 | invasion protein regulator [K] | 8 |  |  |  |  |  |  |  |  |  |  |  |  |
| group_3643 | hypothetical protein (Not in CoGs) | 8 |  |  |  |  |  |  |  |  |  |  |  |  |
| group_667 | hypothetical protein [S] | 8 |  |  |  |  |  |  |  |  |  |  |  |  |
| sinR_3 | HTH-type transcriptional regulator SinR [K] | 8 |  |  |  |  |  |  |  |  |  |  |  |  |
| aaeA_2 | p-hydroxybenzoic acid efflux pump subunit AaeA [V] | 7 |  |  |  |  |  |  |  |  |  |  |  |  |
| alaE_2 | L-alanine exporter AlaE [P] | 7 |  |  |  |  |  |  |  |  |  |  |  |  |
| bphC_1 | Manganese-dependent 2,3-dihydroxybiphenyl 1,2-dioxygenase [E] | 7 |  |  |  |  |  |  |  |  |  |  |  |  |
| butA | Diacetyl reductase [(S)-acetoin forming] [I] | 7 |  |  |  |  |  |  |  |  |  |  |  |  |
| chbP | N,N'-diacetylchitobiose phosphorylase [G] | 7 |  |  |  |  |  |  |  |  |  |  |  |  |
| group_144 | phage tail protein [X] | 7 |  |  |  |  |  |  |  |  |  |  |  |  |
| group_2170 | hypothetical protein [M] | 7 |  |  |  |  |  |  |  |  |  |  |  |  |
| group_2171 | hypothetical protein (Not in CoGs) | 7 |  |  |  |  |  |  |  |  |  |  |  |  |
| group_383 | hypothetical protein [K] | 7 |  |  |  |  |  |  |  |  |  |  |  |  |
| group_391 | hypothetical protein (Not in CoGs) | 7 |  |  |  |  |  |  |  |  |  |  |  |  |
| group_393 | hypothetical protein (Not in CoGs) | 7 |  |  |  |  |  |  |  |  |  |  |  |  |
| group_3977 | Excisionase (Not in CoGs) | 7 |  |  |  |  |  |  |  |  |  |  |  |  |
| group_6038 | transcriptional regulator [K] | 7 |  |  |  |  |  |  |  |  |  |  |  |  |
| group_834 | hypothetical protein (Not in CoGs) | 7 |  |  |  |  |  |  |  |  |  |  |  |  |
| nagX | Amidohydrolase [H] | 7 |  |  |  |  |  |  |  |  |  |  |  |  |
| ridA_1 | Enamine/imine deaminase [J] | 7 |  |  |  |  |  |  |  |  |  |  |  |  |
| ywfH | Bacilysin biosynthesis oxidoreductase YwfH [I] | 7 |  |  |  |  |  |  |  |  |  |  |  |  |
